# Word Entropy-Based Approach to Detect Highly Variable Genetic Markers for Bacterial Genotyping

**DOI:** 10.3389/fmicb.2021.631605

**Published:** 2021-02-03

**Authors:** Marketa Nykrynova, Vojtech Barton, Karel Sedlar, Matej Bezdicek, Martina Lengerova, Helena Skutkova

**Affiliations:** ^1^Department of Biomedical Engineering, Faculty of Electrical Engineering and Communication, Brno University of Technology, Brno, Czechia; ^2^Department of Internal Medicine - Hematology and Oncology, University Hospital Brno, Brno, Czechia

**Keywords:** genotyping, entropy, genetic markers, closely related bacteria, MLST

## Abstract

Genotyping methods are used to distinguish bacterial strains from one species. Thus, distinguishing bacterial strains on a global scale, between countries or local districts in one country is possible. However, the highly selected bacterial populations (e.g., local populations in hospital) are typically closely related and low diversified. Therefore, currently used typing methods are not able to distinguish individual strains from each other. Here, we present a novel pipeline to detect highly variable genetic segments for genotyping a closely related bacterial population. The method is based on a degree of disorder in analyzed sequences that can be represented by sequence entropy. With the identified variable sequences, it is possible to find out transmission routes and sources of highly virulent and multiresistant strains. The proposed method can be used for any bacterial population, and due to its whole genome range, also non-coding regions are examined.

## 1. Introduction

Healthcare-associated infections (HAIs) are a serious worldwide threat with significant impact on mortality and morbidity of hospitalized patients (Arefian et al., 2016). They are defined as infections developed in a hospital or other health care facility that first appear 48 h or more after hospital admission, or within 30 days after having received health care (Haque et al., 2018). Therefore, the ability to identify the source of the infection and monitor the spread of disease is essential. To reach that goal, a process called bacterial typing is used (Ruppitsch, 2016) as it can distinguish individual bacterial strains from one species. However, rapid laboratory and computational techniques' resolution for typing is limited and the ability to distinguish strains among a low diversity hospital bacterial population is practically impossible. Methods such as pulsed-field gel electrophoresis (PFGE) (Schwartz and Cantor, 1984), repetitive sequence-based PCR (rep-PCR) (Skutkova et al., 2019), multilocus sequence typing (MLST) (Maiden et al., 1998), or mini-MLST (Bezdicek et al., 2019) are used to characterize and investigate outbreaks. As housekeeping genes used in MLST or mini-MLST may not have sufficient variability in some cases, there is still a need to develop new approaches to identify variable sequences. One of them is multispacer typing (MST) which uses sequences occurring in junk DNA (intergenic regions and pseudogenes) as they show higher variability than coding genome parts (Foucault et al., 2005). Another approach is single-locus sequence typing (SLST), where one variable sequence identified based on a genome mining approach can have similar discriminatory power to MLST (Scholz et al., 2014). However, if precise analyses of the low diversified bacterial population are required, the only method that can be used is whole-genome sequencing (WGS). For WGS typing, two main approaches exist: single nucleotide variant analysis (SNV) and core genome MLST (cgMLST) or whole genome MLST (wgMLST) (Henri et al., 2017; Schürch et al., 2018), where genes common to bacterial isolates are compared. Nevertheless, the typing methods which require WGS are not suitable for routine clinical practice. Additionally, the comparison of different studies is difficult as different data quality assessment, genome assembly, and results analysis are done (Ruppitsch, 2016).

In this article, we present a new approach to localize highly variable sequences in bacterial genomes of *Escherichia coli* and *Enterococcus faecium*. Detecting new typing fragments is based on a calculation of word entropy in genomes assembled from NGS data. Potential variable sequences are evaluated by phylogenetic analysis, and the obtained results are compared to cgMLST. The core genome was chosen as the identified markers must be presented in all bacterial strains of given species. For that reason, it is not appropriate to study the pan-genome as it contains the core genome and the dispensable genome (Medini et al., 2005). The identified markers will be used for closely related bacterial typing via standard laboratory methods without the need for further WGS. Our approach can be used for any bacteria; the only requirement is aligned isolate genomes.

## 2. Materials and Methods

### 2.1. Dataset

For this article, samples were collected in the University Hospital of Brno. In total, 23 *E. faecium* isolates were obtained from seven hospital departments between 6/2017 and 2/2018 and 21 *E. coli* isolates were collected from a single hospital department between 5/2019 and 7/2019. Therefore, the datasets have different variability rates as they were collected during different periods and from a different number of departments. Sequencing libraries were prepared with KAPA HyperPlus Kits (Roche, Switzerland), and a quality check was performed using a 2100 Bioanalyzer (Agilent Technologies, USA). Libraries were quantified with KAPA Library Quantification kit (Roche, Switzerland). Sequencing was performed on an Illumina MiSeq platform using MiSeq Reagent Kit v2 (500-cycles) (Illumina, USA) and paired-end reads (250 bp) were acquired.

### 2.2. Reference-Based Genome Mapping

After the sequenced data's quality control, the reads were mapped via BBMap (v38.71, Bushnell, 2014) to the human genome (GRCh38.p13) to remove contamination which could emerge during sample preparation. Reads which did not map to the human genome were further analyzed and quality assessment was done. Adapters and low-quality parts of reads were trimmed via Trimmomatic (v0.36, Bolger et al., 2014). The reads length was about 250 bp; thus the reference-based genome mapping approach was chosen, and for this purpose, the Burrows-Wheeler Alignment MEM (v0.7.17-r1188, Li, 2013) was used. The reference sequences were obtained from the GenBank database (Clark et al., 2016). As reference sequence for assembly of *E. faecium* genomes was chosen CP003351.1 (Lam et al., 2012) and *E. coli* genomes were assembled against the sequence BA000007.3 (Hayashi, 2001). Then Samtools (v1.7, Li et al., 2009) was used to remove low-quality and duplicated reads, and consensus sequences were generated.

### 2.3. The Entropy-Based Detection of Variable Sequences

To locate highly variable regions in sequences, the entropy value can be used. The DNA sequences entropy estimation (Schmitt and Herzel, 1997) is based on the Shannon-entropy (Shannon, 1948). In this paper, the entropy calculation modification as inverse value to information content is utilized. This modification commonly used for sequence logo determination (Schneider and Stephens, 1990) measures the conservation of position in bits which better respects different charsets with variable content of ambivalent bases.

Entropy *H*_*i*_ (Schneider and Stephens, 1990) can be calculated as

(1) $$\begin{array}{l} H_{i}=-\underset{a=A,C,G,T}{\sum }f_{a,i}\cdot {\text{log}}_{2}f_{a,i}, \end{array}$$

where *i* is the position in the sequence, *f* is the frequency of one of four nucleotides' occurrence (*a* = A, C, G, T) in position *i* and can be calculated as

(2) $$\begin{array}{l} f_{a,i}=\frac{n_{a}}{n}, \end{array}$$

where *n*_*a*_ is number of occurrences of one nucleotide in one position and *n* is number of all characters at the given position.

In the ideal case, we want to find such a region capable of distinguishing each isolate. For that reason, the current method for entropy calculation in one position (Nykrynova et al., 2019) in the sequence's alignment cannot be used, as it can only distinguish four variants because four nucleotides (A, C, G, T) are presented. In this article, a new unique approach using word entropy is presented, where the word is a nucleotide sequence of defined length.

**Figure 1:**
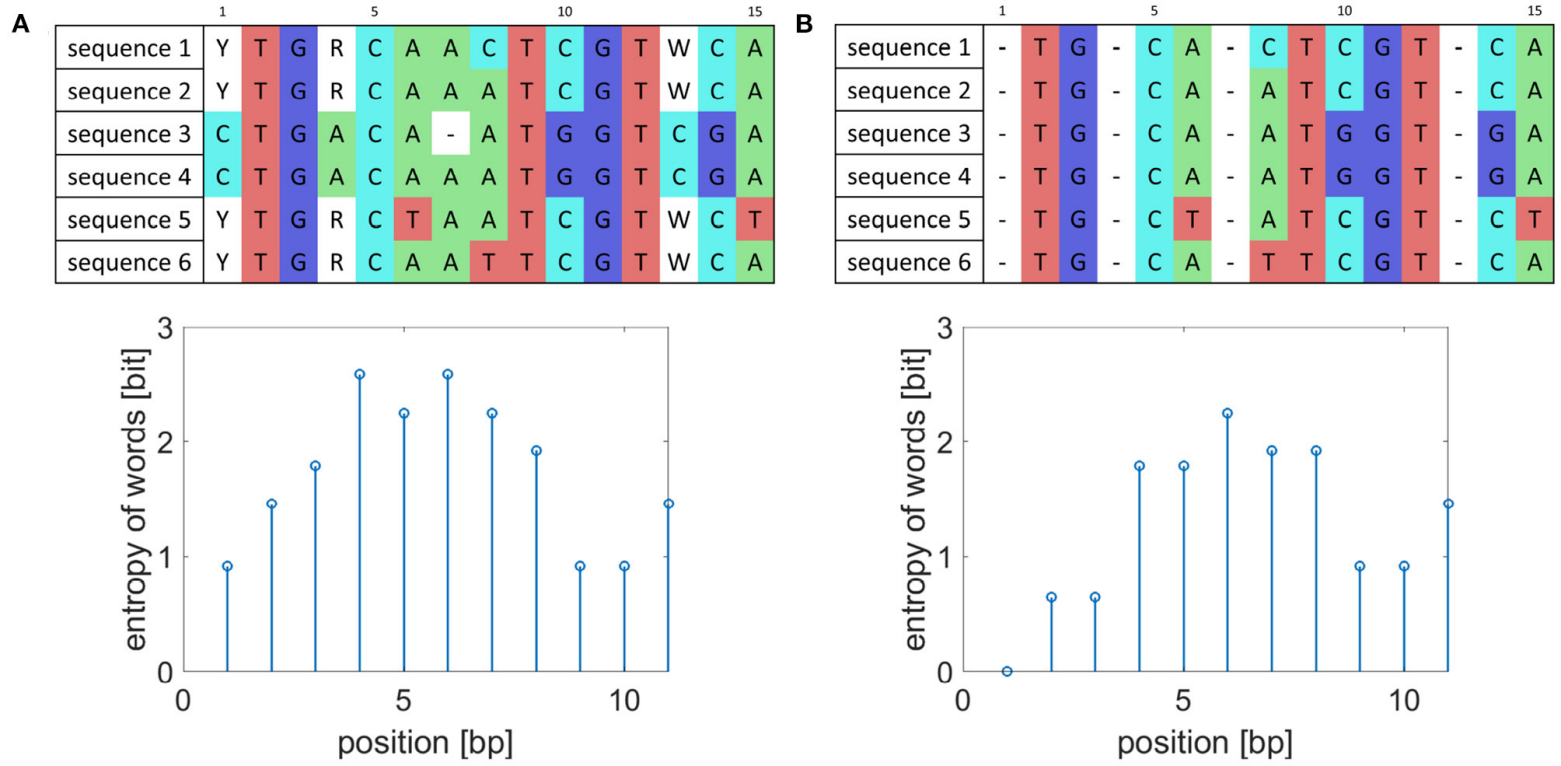
Example of sequence preprocessing. **(A)** Example of aligned sequences before preprocessing and their entropy of word values for words of length five. **(B)** Aligned sequences after preprocessing and corresponding entropy of word values.

As reference-based genome assembly is not perfect during consensus calling ambiguous nucleotides can appear in the consensus sequences, and they artificially increase the entropy value as shown in Figure 1. To prevent detecting falsely variable sequences, the whole columns of aligned sequences where ambiguous nucleotides occur were removed and to preserve the original length of the created consensus sequences replaced by gaps. Also, the detected markers should occur in all analyzed isolates. Thus, when gaps appear in some sequences, the gaps represented by dashes are added to the whole columns as is also shown in Figure 1.

**Figure 2:**
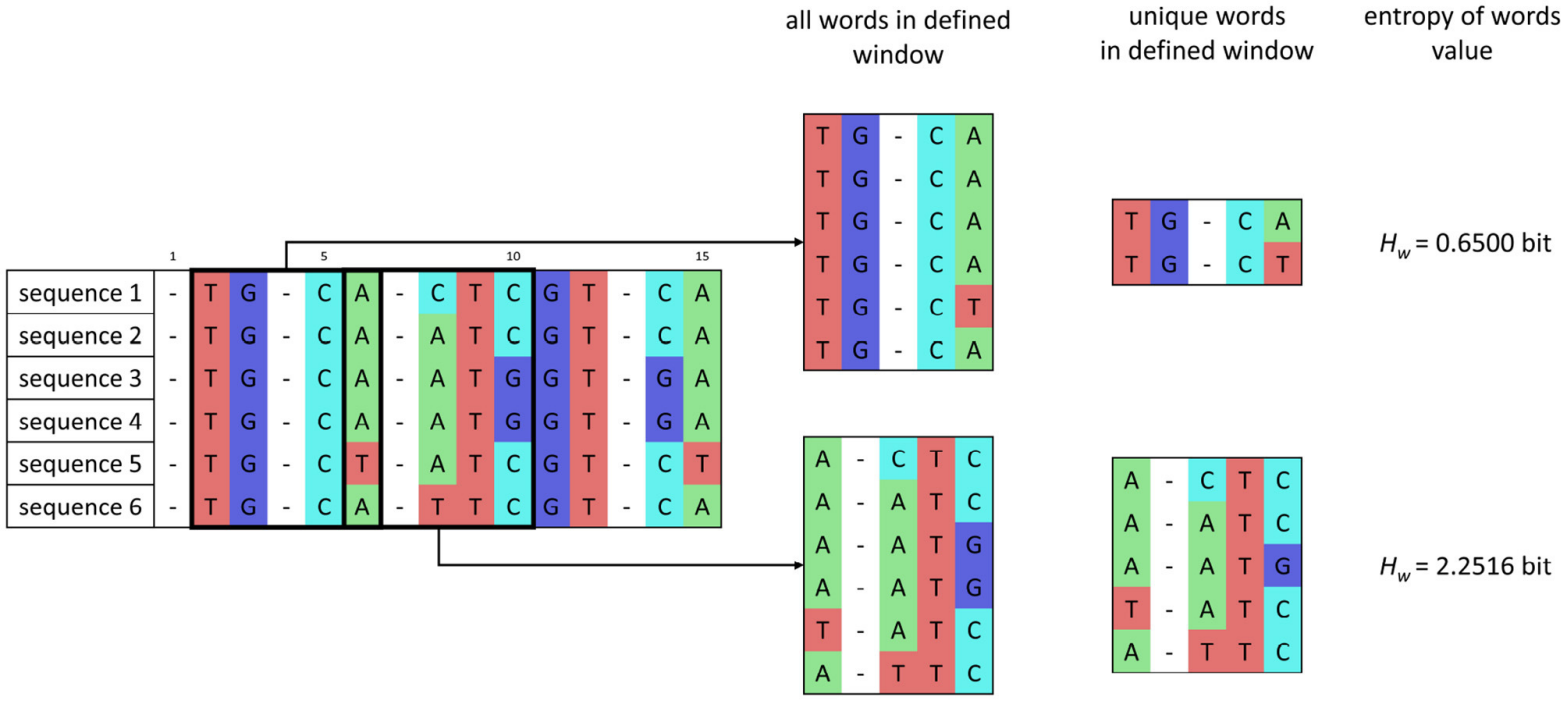
Scheme of entropy of words' calculation for a set of sequences after preprocessing and for word length of 5 bp and one nucleotide window shift.

In the window *w*, whose size corresponds to word length, the number of unique words is determined, and then the entropy of words *H*_*w*_ is calculated as

(3) $$\begin{array}{l} H_{w}=-\sum \left(\frac{k_{w}}{n}\cdot \left({\text{log}}_{2}\frac{k_{w}}{n}\right)\right), \end{array}$$

where *k*_*w*_ is the number of occurrences of unique words in each alignment in the current window, and *n* is the number of isolates. The sum is applied over the alignment in the current window. After estimating the entropy value, the window moves with a shift of one nucleotide. The principle of entropy of words' calculation is shown in Figure 2.

For defined sliding window sizes *w* (also called word length), the entropy signal with length *N* − *w* + 1 is obtained, where *N* is the analyzed genome's length.

In the signal, the maximum value is identified. In theory, the maximum value can be defined as a binary logarithm of the number of sequences. Based on the maximum value that occurs in the signal, the threshold is set as a maximum value reduced by 0.4 bit, and signal peaks above the threshold are labeled as potential genetic markers. The stated value was chosen empirically based on the number of potential variable regions to ensure that some regions will have sufficient discriminatory power; however, it can be adjusted to analyzed data by the user. The aligned sequences corresponding to peaks are extracted and analyzed. If two peaks are distant less than the window length, the sequences corresponding to the peaks are merged and analyzed as one. Developed source codes and functions in Matlab R2020a can be found at https://github.com/marketanykrynova/markers_detection.

### 2.4. Phylogenetic Analysis

In the first part, each set of aligned sequences corresponding to peaks was analyzed. The evolutionary distances were calculated based on the Kimura (Kimura, 1980) model, which is used as standard. From obtained distances, the phylogenetic trees were constructed based on UPGMA. Cluster analysis was conducted, and the clusters from the trees were established and compared to results obtained from cgMLST. The threshold for clusters determination was set to intersect the tree so that the maximum number of clusters wasobtained.

The results showed that no set had sufficient discriminatory power to distinguish between strains. That is why the combination of two aligned sequences sets was used. The identified variable sets of sequences were merged, which created one long sequence for each analyzed bacterial isolate. Then for merged sequences, again the evolutionary distances were calculated and based on them, the phylogenetic trees were constructed and examined. The length of a variable set of sequences had a proportional impact on the created phylogenetic tree. If the sequences in one variable set had a longer length, they had a higher impact on the tree and vice versa, the set of shorter sequences did not affect the final tree so much. Nevertheless, sequences variability has the main impact on the phylogenetic tree.

## 3. Results and Discussion

### 3.1. Sequence Type Determination and cgMLST Analysis

The incorporated *E. coli* and *E. faecium* cgMLST, cgSNV, and wgSNV schemes in Ridom SeqSphere+ software (Ridom, DE) were used to analyze the genetic relatedness of sequenced bacterial genomes. For *E. coli*, this included 3,152 target genes used to generate a cgMLST dendrogram. For the cgSNV minimum spanning tree analysis, 152,212 aligned nucleotide sites were analyzed. In total, 176,629 aligned nucleotide sites were used for wgSNV minimum spanning tree analysis. For *E. faecium*, the scheme included 1,423 target genes for cgMLST, 7,707 aligned nucleotide sites for cgSNV and 10,858 aligned nucleotide sites for wgSNV analysis. The WGS data were used to determine sequence types (STs) of both, *E. coli* and *E. faecium* strains. For *E. coli*, seven housekeeping genes (*adk, fumC, gyrB, lcd, mdh, purA, recA*) and for *E. faecium* again seven housekeeping genes (*atpA, ddl, gdh, purK, gyd, pstS, adk*) were analyzed. For *E. faecium* three STs were identified (4 × ST 117, 1 × ST 18, 18 × ST 80), and for *E. coli* eleven STs were recognized and one ST remains unknown (1 × ST 69, 4 × ST 131, 1 × ST 95, 2 × ST 404, 2 × ST 38, 2 × ST 1049, 4 × ST 58, 1 × ST 297, 1 × ST 517, 2 × ST 101, 1 × ST UNW). The complete results are attached as Supplementary File 1.

### 3.2. Potential Genetic Markers Analysis

Sequences with a high value of word entropy were identified for word lengths from 50 to 400 bp with a step of 50 bp. Traditional laboratory methods can use identified markers of length from this range. In the first part, one variable fragment with high word entropy values was used to distinguish the strains of *E. coli* and *E. faecium*. Examining 1,358 fragments with high variability for all word sizes showed that the maximum number of clusters that can be distinguished for *E. coli* was 13. For *E. faecium*, 104 potential genetic markers for all word sizes were analyzed, and the maximum number of distinguishable clusters was 5. The complete results are in Supplementary File 2.

The number of distinguishable clusters using one variable fragment is not high enough. For that reason, two variable fragments with word entropy above threshold were analyzed. For all combinations of two possible genetic markers, the phylogenetic trees were constructed, and the number of clusters was calculated. In the next step, the potential genetic markers with the highest number of clusters were analyzed. For all word sizes, 136,661 combinations of possible genetic markers for *E. coli* and 685 for *E. faecium* were examined in total.

In case of need, the combination of even more markers can be used; however, the time of analysis will grow as the time complexity is *O*(*n*^*m*^), where *m* is the number of variable fragments.

**Figure 3:**
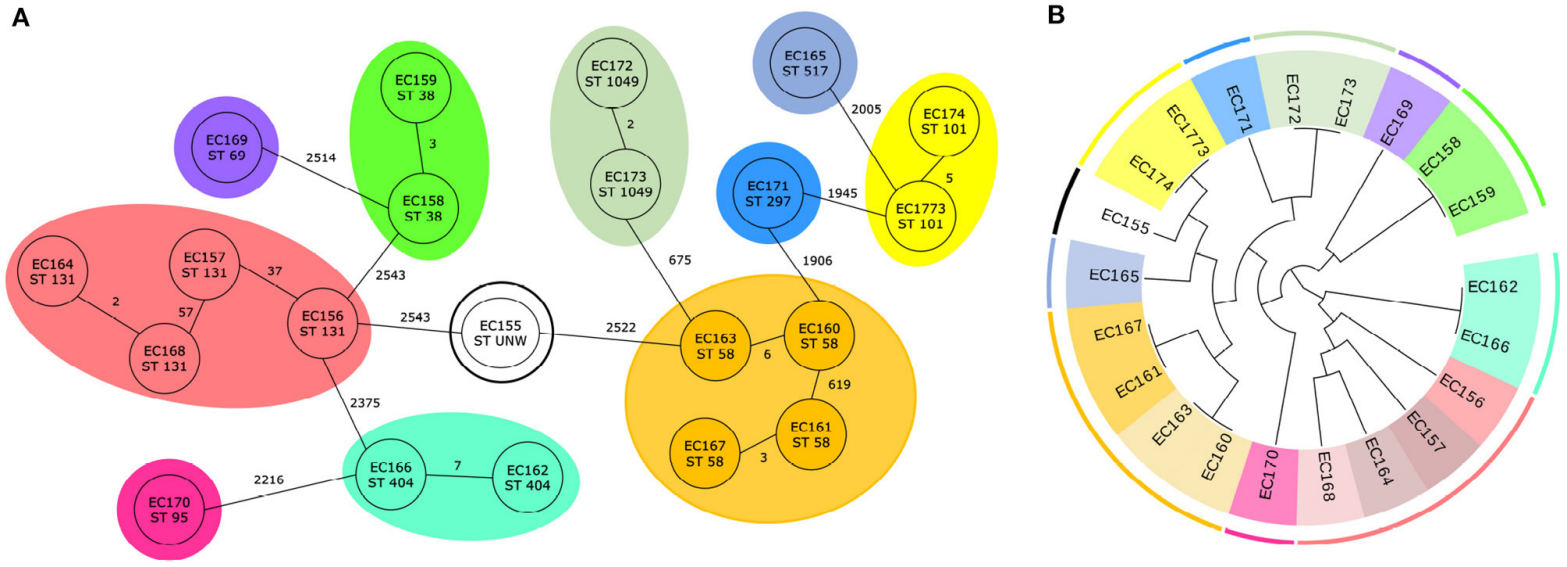
Classification of 21 *E. coli* genomes. **(A)** Core genome MLST analysis of 21 *E. coli* isolates with predefined marked clusters (outer circles) and sequence types (inner circles). **(B)** Cladogram of 21 *E. coli* isolates based on two identified genetic markers with highlight clusters obtained from core genome MLST analysis (outer circle) and subgroups obtained based on analysis of identified variable sequences (inner circle) created by Evolview (Subramanian et al., 2019).

For *E. coli*, 29 trees were examined which divided the isolates to 15 clusters. Ten trees classify the genomes according to the 11 predefined clusters from cgMLST. The results from cgMLST with 11 marked sequence types and 11 defined clusters and a cladogram created based on the combination of two genetic markers with 15 labeled clusters are shown in Figure 3.

In each case for one genetic marker, the region which contained the genes for hypothetical protein (NP_308436.2) and S-formylglutathione hydrolase (NP_308437.1) was selected. In five out of ten cases, the intergenic region in front of hypothetical protein was also incorporated. As the second genetic marker, two regions were most often (three times) chosen. The first one contained outer membrane protein (NP_313225.1) and in one case the intergenic region in front of the gene too. The second one started in fimbrial-like adhesin protein (NP_310944.1) and ended in hypothetical protein (NP_310945.1). Four other genetic markers were selected only one time. Two of them consisted of non-coding region and gene [PhoH family P-loop ATPase (NP_309293.1) in the first case, excinuclease UvrABC subunit UvrC (NP_310678.2) in the second case], for another marker part of the gene valyl-tRNA synthetase (NP_313262.1) was picked, and in the last case, as a genetic marker the non-coding region was selected. Detailed information is available in Supplementary File 2 and all phylogenetic trees that correctly classified genomes are depicted in Supplementary Figure 1.

**Figure 4:**
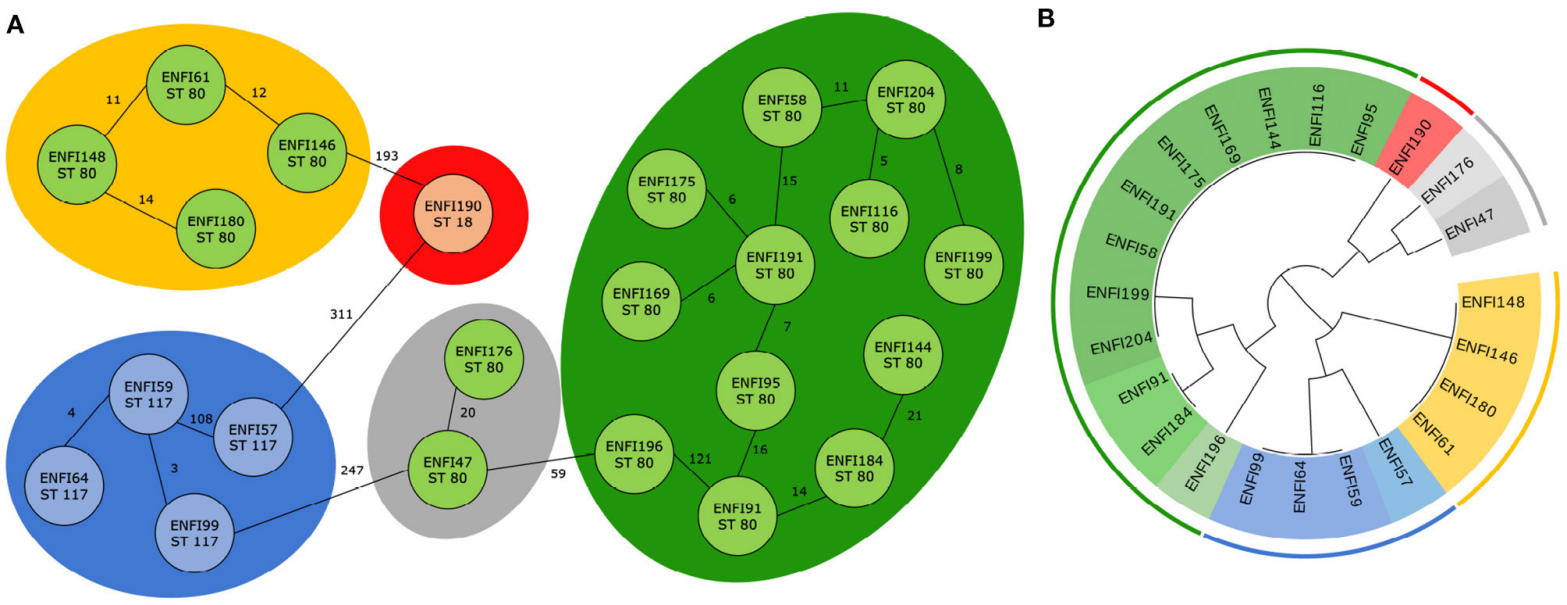
Classification of 23 *E. faecium* genomes. **(A)** Core genome MLST analysis of 23 *E. faecium* isolates with predefined marked clusters (outer circles) and sequence types (inner circles). **(B)** Cladogram of 23 *E. faecium* isolates based on two identified genetic markers with highlight clusters obtained from core genome MLST analysis (outer circle) and subgroups obtained based on analysis of identified variable sequences (inner circle) created by Evolview (Subramanian et al., 2019).

For *E. faecium*, 22 trees which divided the isolates into nine clusters were examined. It was found that six trees classify the genomes according to five predefined clusters from cgMLST. In Figure 4, the results from cgMLST analysis with three labeled sequence types, five clusters and a cladogram constructed based on the identified combination of two genetic markers with nine labeled clusters shown.

As in the previous case, in all possible combinations, one genetic marker always remained the same, and it was RNA methyltransferase (WP_002294889.1). In one of six cases, the intergenic region around the gene was also incorporated into the variable genetic part. Mg-translocating P-type ATPase (WP_002304253.1) was in most cases (three times) selected as the second marker. The region that contained the gene for DUF1430 domain-containing protein (WP_002288541.1) was chosen twice. For the last possible genetic marker, the gene for nucleoside-diphosphate kinase (WP_002293327.1) was picked. The complete results are available in Supplementary File 2 and all phylogenetic trees correctly classifying genomes are shown in Supplementary Figure 2.

### 3.3. Word Sizes

**Figure 5:**
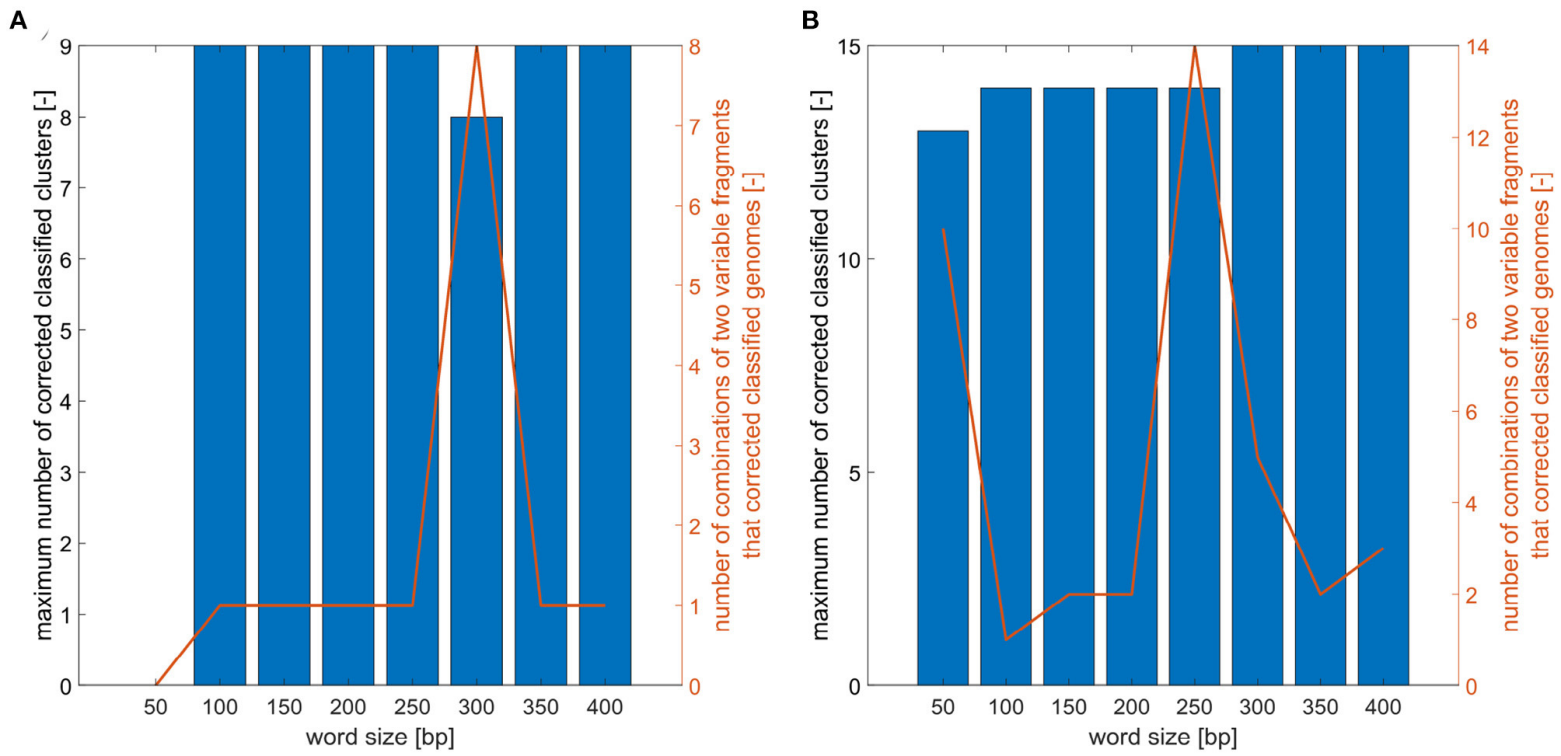
Analysis of word size impact on number of correctly classified clusters. **(A)** Maximum number of correctly classified clusters for combinations of two variable fragments for different word sizes and number of two variable fragment combinations that corrected classified analyzed *E. faecium* isolates. **(B)** Maximum number of correctly classified clusters for combination of two variable fragments for different word sizes and number of two variable fragment combinations that corrected classified analyzed *E. coli* isolates.

For defined word sizes, a maximum number of correctly classified clusters for phylogenetic trees based on two marker combinations was established, as is shown in Figure 5. For analyzed trees the number of clusters was determined and the correct genomes classification was controlled. The results showed that for *E. faecium* the word lengths do not significantly affect the maximum number of correctly established clusters, which is nine for all word sizes except the length 50 and 300 bp. On the contrary, the resolution power for *E. coli* grows with increasing word lengths and the best results (distinguish 15 clusters) can be obtained for a window of size 300 bp and more.

### 3.4. Results Verification

The analyzed datasets were extended by complete genomes sequences of *E. coli* and *E. faecium* obtained from the GenBank database. For each bacterium, six sequences were downloaded, and the STs were determined. For *E. coli* isolates (CP050212.1, CP050219.1, CP050211.1, CP050201.1, CP050214.1, CP050205.1) five STs were identified (1 × ST 2705, 1 × ST 2280, 1 × ST 70, 1 × ST 602, 2 × ST 131), and for *E. faecium* isolates (CP040236.1, CP036151.1, CP027501.1, CP035666.1, CP035660.1, CP035220.1) one ST was recognized, and one ST remains unknown (5 × ST 80, 1 × ST UNW). The results are in Supplementary File 1. Using the BLAST+ (v2.6.0+, Camacho et al., 2009) the identified variable markers were located in these genomes. The markers sequences for both sets (original and database) were analyzed together. The sequences were aligned, evolutionary distances were calculated, and phylogenetic trees were constructed. Totally, 27 isolates of *E. coli* belonging to nine STs were classified to 17 clusters and 29 isolates of *E. faecium* belonging to four STs were five times classified to 11 clusters and once to 10 clusters. All created phylogenetic trees are depicted in Supplementary Figure 3.

The identified genetic markers can be used to typing novel isolates. However, if a large number of new isolates should be analyzed, the algorithm should be run again to find markers with the highest discriminatory power for the analyzed bacterial population.

### 3.5. Analyzed Bacteria

The proposed approach was tested on two bacteria—*E. faecium* and *E. coli* which belong among significant HAIs pathogens. *E. faecium* is a Gram-positive bacterium and can be a source of nosocomial infections, especially in immunocompromised patients, and causes endocarditis, bacteremia or urinary tract infection. In addition, some *E. faecium* isolates can develop drug resistance to several antibiotics groups such as glycopeptides, beta-lactams, fluoroquinolones, or aminoglycosides (Yoong et al., 2004; Castillo-Rojas et al., 2013). Another pathogen from Gram-negative bacteria is *E. coli*, which can be found in human intestinal flora as a commensal. However, as an opportunistic pathogen *E. coli* represents a huge public health problem. It is one of the most common causes of HAIs typically connected to meningitis, pneumonia, urinary tract infections, and soft tissue infections (Jaureguy et al., 2008). *E. coli* strains have the ability to accumulate resistance genes; thus, the resistance to broad-spectrum cephalosporins, carbapenems, aminoglycoside, fluoroquinolones, and polymyxins is often observed (Poirel et al., 2018).

## 4. Conclusion

As typing is crucial for identifying the source of infection and outbreak monitoring, it is necessary to have a highly sensitive typing method to distinguish closely related bacterial strains. However, currently used methods have insufficient discriminatory power except whole genome sequencing. Nevertheless, WGS is time-consuming, and data processing is not possible in routine clinical practice. For this reason, a new approach to detect variable genetic markers from WGS data is presented. The WGS is only used once to obtain the first set of data for genetic markers detection. To locate highly variable sequences, word entropy is employed. The located markers can be used for typing a closely related bacterial population via standard laboratory methods. Thus, another genome sequencing will be necessary only if a large number of new isolates should be analyzed.

Using the entropy-based approach, new genetic markers with the same or even higher discriminatory power than cgMLST can be identified. As the length of words from 50 to 400 bp with a step of 50 bp is applied, potential markers can be used in standard laboratory techniques because they have suitable length. The only requirement for the proposed approach is that genomes must be aligned, which can cause a problem mainly when large genomes should be examined. The presented method was tested on both Gram-positive and Gram-negative bacteria.

As in both tested datasets, one marker with sufficient discriminatory power for typing of low diversity bacterial population was not found, the combination of two markers was employed. Based on two identified markers, we were able to distinguish the Gram-negative and Gram-positive bacterial population at the same and even a higher discrimination level than via classic laboratory methods using only two variable sequences instead of seven housekeeping genes. The identified markers will be used in routine laboratory methods for bacterial typing. The low number of variable fragments that should be examined will shorten the time of analysis.

Commonly used typing methods mainly use coding regions and analyze genes conserved and presented in all isolates of the given species. Hence the variability rate is lower and typing the closely related population is not possible. The proposed method uses the whole genome range; thus, also intergenic genome regions are analyzed as they have higher variability than coding areas and can contain genetic markers for typing related isolates. Based on identified markers, the closely related bacterial population can be diversified and to find out the transmission routes and source of outbreaks will be possible.

## Data Availability Statement

The datasets presented in this study can be found in online repositories. The names of the repository/repositories and accession number(s) can be found at: https://www.ncbi.nlm.nih.gov/, PRJNA675431.

## Author Contributions

MN, VB, KS, and HS contributed to the conception and design of the study. MN implemented the algorithm and evaluated the results. ML and MB ensured the biological aspects of the project. All authors read and approved the final manuscript.

## Conflict of Interest

The authors declare that the research was conducted in the absence of any commercial or financial relationships that could be construed as a potential conflict of interest.

## Supplementary Material

Supplementary File 1 MLST analysis results for *E.coli* and *E. faecium*.

Supplementary File 2 Potential and final genetic markers for *E. coli* and *E. faecium*.

Supplementary Figure 1 Phylogenetic trees that correctly classified *E. coli* genomes with marked clusters.

Supplementary Figure 2 Phylogenetic trees that correctly classified *E. faecium* genomes with marked clusters.

Supplementary Figure 3 Phylogenetic trees of original and database genomes of *E. faecium* and *E. coli* genomes with marked clusters.
